# Microfluidic on-chip biomimicry for 3D cell culture: a fit-for-purpose investigation from the end user standpoint

**DOI:** 10.4155/fsoa-2016-0084

**Published:** 2017-03-02

**Authors:** Ye Liu, Elisabeth Gill, Yan Yan Shery Huang

**Affiliations:** 1 Department of Engineering, University of Cambridge, Trumpington Street, Cambridge, UK, CB2 1PZ

**Keywords:** 3D culture, complexity, microenvironment, microfluidics, organ-on-chip

## Abstract

A plethora of 3D and microfluidics-based culture models have been demonstrated in the recent years with the ultimate aim to facilitate predictive *in vitro* models for pharmaceutical development. This article summarizes to date the progress in the microfluidics-based tissue culture models, including organ-on-a-chip and vasculature-on-a-chip. Specific focus is placed on addressing the question of what kinds of 3D culture and system complexities are deemed desirable by the biological and biomedical community. This question is addressed through analysis of a research survey to evaluate the potential use of microfluidic cell culture models among the end users. Our results showed a willingness to adopt 3D culture technology among biomedical researchers, although a significant gap still exists between the desired systems and existing 3D culture options. With these results, key challenges and future directions are highlighted.

## Lay Abstract

This article summarizes the progress in the development of microfluidics-based 3D cell/tissue culture models to date, including organ-on-a-chip and vasculature-on-a-chip capable of mimicking *in vivo* tissue architecture. Specific focus is placed on addressing the question of what kinds of 3D culture and system complexities are deemed desirable by the biomedical community. A research survey targeting biomedical researchers was designed to evaluate the potential application of microfluidics-based 3D culture systems and a positive attitude was recorded among its participants.

Human combat against lethal diseases such as cancer and age-related deterioration has long been hampered by a lack of effective, accessible and safe therapies. This can be partly attributed to the inefficiency of current drug development practice [1], especially the low-predictive power of preclinical models [2–4]. Traditionally, drug screening starts in 2D petri dishes where immortalized human cell lines are cultured in monolayers. Though easy for standardization, the petri dish system is too simplistic and lacks microenvironment complexity and physiological relevance to living tissues. Too often, 2D models produce false positive results, giving unreliable or even misleading predictions for down-stream tests [5]. Physiologically relevant conditions can be found in animal-based systems such as *ex vivo* chicken chorioallantoic membrane assays, *in vivo* transgenic mice or mouse xenografts [6]. However, drugs that work well in an ancmal model often fail when translated in humans [7,8], due to the phylogenetically remote relationship between different species. In addition, animal studies have long been criticized for their high cost, labor-intensiveness, low reproducibility and poor controllability over physiological parameters [9]. All these factors give rise to the emergence of 3D culture technology, which aims to create a more controllable yet accurate culture platform for preclinical study. Figure 1 summarizes a number of established preclinical models with varying complexity for drug development.

**Figure 1.**
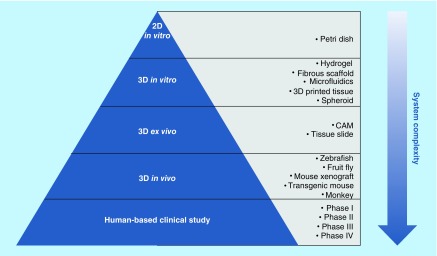
**Preclinical and clinical models for drug development.** With an increase in system complexity, preclinical models range from 2D petri dish, to 3D *in vitro* and *ex vivo* culture systems, to *in vivo* animal models. Clinical studies are based on human subjects and consist of four testing phases. CAM: Chicken chorioallantoic membrane assay.

3D cell culture, as its name suggests, refers to the *in vitro* culture of human cell lines or patient-derived tissues in a more physiologically relevant, 3D miniature niche [10]. Recent advances in this field include the design of extracellular matrix (ECM) mimicking hydrogel matrices [11,12] and fiber scaffolds [13,14], microfluidic ‘organ-on-a-chip’ [15,16], self-assembled multicellular spheroids [17,18] and bioprinted micro tissues [19,20]. Table 1 lists some of the key microenvironment cues, and the reported methods to replicate them in 3D culture models. It is expected that by increasing the structural, chemical and physical complexity of the *in vitro* culture environment, 3D cell culture will possess great advantages over the conventional 2D culture and may demonstrate increased predictive power as a next-generation drug testing platform [21]. Moreover, since 3D culture niches accommodate human cells/tissues, they can potentially help bridge the gap between animal studies and human-based clinical trials [22]. With the drive toward drug screening, many of the aforementioned 3D culture designs have been commercialized into scalable multiwell plate format. Table 2 summarizes examples of some of the currently available 3D culture systems, ranging from scaffold-based models such as 3D hydrogels and polymeric membranes to scaffold-free ones such as microfluidics, spheroids and direct-printed mini tissues.

**Table 1.** **Functions of microenvironment cues and biomimetic examples in various 3D culture systems.**

**Biomimetic microenvironment cues**	**Function**	**3D hydrogel**	**Polymer scaffold**	**Microfluidics**	**Spheroid**	**3D printing**
Spatial organization of cells/ECMs	Reproduce 3D tissue structure	[23,24]	[25,26]	[27,28]	[29]	[30,31]
Chemical gradient	Reproduce gradient of signaling molecules/growth factors	[32,33]	[34]	[35,36]	[37]	–
coculture of heterotypic cells	Enable cell–cell cross-talk	[38,39]	[40,41]	[42–44]	[45,46]	[47,48]
External force stimuli	Enable biomechanical sensing	[49,50]	[51,52]	[53,54]	[55]	[56,57]
ECM	Reproduce structural, mechanical and chemical properties of ECM	[58,59]	[60,61]	[62,63]	[64,65]	[66,67]
Hypoxia	Induce tumor angiogenesis	[68]	[69]	[69–70]	[64]	[71]
Topography	Direct cell motion and confine cell distribution	[72,73]	[60,61]	[74,75]	[76]	[77,78]
Simulated body fluids	Reproduce blood/interstitial flow	–	–	[79–81]	–	–

Typical microenvironment features include spatial organization of cells/ECM, biochemical gradient, co-existence of heterotypic cells, external mechanical stimuli, ECM, hypoxia, topography and body fluids. Individual or a combination of microenvironment cues can be replicated using various 3D culture methods such as hydrogel matrix, fibrous polymer scaffold, microfluidics, multicellular spheroid and 3D printed tissue.

ECM: Extracellular matrix.

**Table 2.** **Characteristics of commercially available 3D culture systems.**

**3D culture strategy**	**Commercialized product**	**Main feature**	**Maximum culture time**	**Chemical gradient**	**Flow**	**coculture**	**ECM**	**Notes**	**Ref.**
Scaffold-based culture	Biomimesys	Cells on top of hyaluronic acid scaffolds; 96-well format	21 days	No	No	No	3D hyaluronic acid-based hydrogel	Highly porous gel (150–200 μm pores); Non-uniform spheroids (50–250 μm)	[96,97]
	LiverChip	Porous membrane separating two compartments; 12-well format	14 days	No	On-chip micropump-controlled recycling perfusion	Heterotypic cells on opposite sides of a membrane	Polymer membrane mimicking liver sinusoid architecture	Limited to liver organ	[98,99]
Microfluidic chips	OrganoPlates (by MIMETAS)	Microfluidics; 384-well format	15 days	Controllable gradient	Perfusion controlled by Phase guide technology	Heterotypic cells in different regions of microchannels	3D hydrogel insertion	Formation of microvasculature (diameter <100 μm)	[100,101]
	SynVivo	Individually packed microfluidic chips	10 days	Controllable gradient	External pump-controlled perfusion	Heterotypic cells/tissues in different regions of microchannels	2D coating of hydrogel/ECM molecules	Linear/bifurcating channels and tortuous networks (diameter >100 μm)	[102,103]
Scaffold-free cell aggregates	InSphero	Self-assembled multicellular spheroids; 96-well format	Weeks	Gradient as a function of depth within spheroids	No	Heterotypic cells aggregated in spheroids	Endogenous ECM	Tight junction; Controllable spheroid size; Limited by cells’ ability to form spheroids	[104,105]
	ExVive3D (by Organovo)	Layer-by-layer bioprinted tissues; available in multiwell format	28 days	Gradient as a function of depth within micro tissues	No	Controllable spatial distribution of heterotypic cells	Endogenous ECM	Tight junction; Controllable tissue size; Wide choice of cell types	[106,107]

Two categories of 3D culture systems are commercially available. Scaffold-based culture models use 3D hydrogel matrixes or polymeric membranes/scaffolds as miniature cell culture niches. Scaffold-free culture can be realized by microfluidics, self-assembled spheroid or direct-printing technologies. Each 3D culture method is able to reproduce a set of microenvironment features.

ECM: Extracellular matrix.

With the rapid development of an extended range of enabling technologies for 3D cell culture, one question desperately needs to be answered: what determines an ideal 3D culture model? We believe at least three criteria should be considered: balanced complexity and user-friendliness, proven functionality and high controllability. First, an *in vitro* model needs to demonstrate considerable complexity in order to reproduce some of the key attributes of the natural cellular microenvironment. These elements, as summarized in Figure 2, include biomechanical stimuli, extrinsic forces, chemical gradients and cell–cell interactions. However, it is worth mentioning that the choice of the system and complexity always depends on the research question to be addressed. Second, in order to add value to basic science and clinical studies, a 3D culture model should demonstrate its physiological relevance, showcasing its capability to recapitulate key characteristics of *in vivo* scenarios and key functionalities of tissues/organs. Furthermore, a culture system has to be robust and highly controllable to facilitate standardization, high throughput and commercialization.

**Figure 2.**
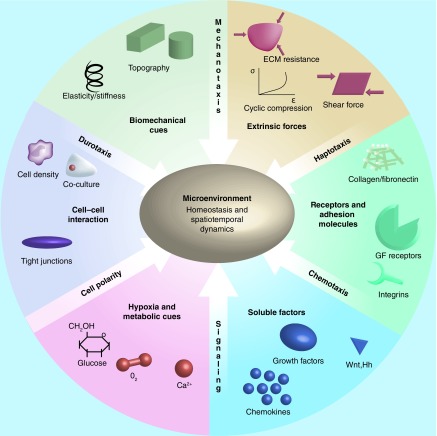
**Key microenvironment homeostasis and spatiotemporal dynamics that are desirable to be modeled in *in vitro* 3D culture systems.** All microenvironment cues are dynamic, heterotypic and drivers of cell behavior and fate. ECM: extracellular matrix; GF: growth factor. Adapted from [82,83].

In this article, we will pay specific focus on addressing the question of what kinds of system complexities are deemed desirable by the biological and biomedical community. We first review the microfluidics-based 3D culture technology for organ-on-chips and *in vitro* microvascular models. Then we present the results obtained from a questionnaire designed to evaluate the potential use of microfluidic cell culture among biomedical researchers. Key challenges and future direction for microfluidic 3D culture are also highlighted.

## On-chip biomimicry

Microfluidics refers to the manipulation of fluids of small volume (usually less than 10 μl) inside a channel of sub-millimeter scale. At micro scale, various phenomena are prevalent in fluids: laminar flow, improved molecular diffusion and accelerated heat transfer. In the early 1990s when the first microfluidic chips were fabricated in glass or oxidized silicon, they were primarily used in microelectromechanical systems [84] and separation science [85]. Later, microfluidics were introduced to the biomedical community because of their potential to greatly reduce the consumption of expensive reagents and valuable samples. Another attractive attribute of microfluidics is that laminar flow enables the design of physiologically relevant fluidic patterns, chemical gradients and spatially organized cells/ECMs. From the mid-1990s, the concept of on-chip biomimicry of tissues/organs using microfluidics-based miniature chips slowly began to prosper. Among the earliest attempts, Frame *et al*. designed branching micro tubes (20–50 μm in diameter) etched on a borosilicate microscope slide glass to reproduce the geometry of the arteriolar microcirculation [86]. Endothelial cells were cultured inside the microchannel under perfusion of culture medium for up to 24 h. However, the microfluidic chips made of glass/silicon were brittle, the fabrication procedure was slow and required a clean room facility. Therefore, scientists started to prototype microfluidic chips using a more robust and low-cost elastic polymer called polydimethylsiloxane (PDMS) [87]. This move from glass/silicon to PDMS drastically accelerated the application of microfluidics in biology. PDMS chips are relatively easy to fabricate, and are able to generate statistically analyzable information in parallel operation. Also of critical importance, the optical transparency of PDMS enables real-time imaging, which is desirable for almost all branches of biomedical research. Over the past decade, a myriad of microfluidic designs with complex microarchitecture, significant bioelectrical and biochemical functionality have been realized through the addition of pneumatic valves [88], pillars [89], droplets [90], electrodes [91], hydrogel matrices [92] and electrospun fiber scaffolds [63,93]. Such delicate microfluidics platforms have the potential to be developed for 3D biomimetic tissues and organotypic cultures, for a range of biomedical purposes. One popular example is the microfluidic-based organ-on-a-chip systems [94,95], some of which are illustrated in Table 2.

Probably one of the most famous examples of microfluidic organ-on-a-chip is the lung-on-a-chip realized by Huh *et al*. to mimic pulmonary edema [53]. Briefly, an upper air channel was separated from a lower liquid channel by a porous PDMS membrane, the opposite sides of which formed epithelium and endothelium layers respectively. Two side channels parallel to the central channels were connected to a vacuum pump that cyclically mimicked the breathing motion of the lung. This lung-on-a-chip is a hallmark achievement in organ-on-chip development and has inspired the development of other human organ chips such as the gut-on-a-chip [108], kidney on-a-chip [109] and placenta on-a-chip [62]. More complex microsystems have also been created for the ‘human-on-a-chip’, in which various organ equivalents are integrated into one single chip. For example, a four-organ-chip was established by Maschmeyer *et al*., demonstrating long-term coculture of liver spheroids, an intestine barrier insert, a skin biopsy insert and a polymeric kidney membrane. The four biomimetic organs were interconnected through a microfluidic circulation system perfused with surrogate blood [110]. Such a multi-organ platform can be used to observe the systemic responses of intercommunicating organs during complex physiological and pathophysiological processes including food metabolism and pharmacokinetics. Apart from culturing human organ-specific cell lines in microfluidic chips, other cell and tissue sources such as human tissue slides and pluripotent stem cells have also been reported. For instance, organotypic culture of brain slices for up to 40 days were performed in a microfluidic system which enabled the compartmentalization of different functional units of the brain as well as easy manipulation of selected neural circuits [111]. Stem cell culture using microfluidics was also demonstrated in a study on ECM-mediated neural stem cell differentiation [112]. Finally, it is worth mentioning that the microenvironmental cues for these biopsy-derived tissues or self-organizing stem cells have to be well understood and carefully designed to ensure long-term culture as well as well-controlled cell fate [113].

## Microfluidic vasculature models

Aside from microfluidic organ-on-a-chips, another key focus of tissue engineers is blood vessels and capillaries, which are the basic building blocks of almost all organs and many important tissue structures such as the blood–brain barrier [114], lung alveoli [115] and renal glomerulus [116]. Vasculature enables gas exchange in the body as well as the transport of nutrients, waste, pathogens, blood cells and circulating cancer cells. Lack of proper vascularization can induce the formation of necrotic cores [117], which has been a common problem in many large-scale tissue constructs. Of equal importance, vascular endothelial cells are active in many signaling events such as hypoxia-initiated angiogenesis [118], stem cell differentiation [119] and the insulin signaling pathway [120]. Many cell–cell interactions that take place *in vivo*, including leukocyte–endothelium [121] and platelet–endothelium interaction [122], also involve the active participation of vascular endothelial cells. Vasculature is indispensable in modeling any process that is circulation driven such as inflammatory responses [123], cardiovascular diseases [124], cancer intravasation [125] and extravasation [126]. Therefore, creating 3D *in vivo*-like vasculature has always been an important research question in the engineering of tissue surrogates and disease-on-a-chip.

Thanks to the many advantages microfluidics possess, tissue engineers have been employing microfluidics technology to mimic vasculature networks over the past two decades [127,128]. First, microfluidic channels, from a few to hundreds of microns in diameter, are a convenient tool to mimic the geometry of microcirculation. Second, flow in microfluidic devices can be tailored to recapitulate the dynamic and fluidic microenvironment that cells experience *in vivo* including body fluids (blood and interstitial fluids), shear stress and mass transportation. One successful example is an endothelialized microvasculature developed by Tsai *et al*., with branching channels (smallest diameter of 30 μm) emulating a postcapillary venule. Using this hemodynamics-mimicking platform, they studied the effect of an inflammatory cytokine TNF-α and the drug hydroxyurea on microvascular obstruction under perfusion of patient blood [129]. Apart from the simulation of vasculature anatomy and hemodynamics, spatiotemporally defined chemical gradients are also achievable in microfluidic devices by manipulating liquids of different chemical concentrations in parallel channels. Third, heterotypic cells can be subsequently seeded and cocultured in the same microfluidic chip. Such coculture capability offers significant convenience for researchers interested in cell–cell interactions [130] happening in tumor-immune response [131] and vasculogenesis [132]. To summarize, microfluidics provide unique microenvironmental attributes such as lumen architecture, flow and spatial distribution of cells/particles that are difficult to define using other 3D culture technologies. Therefore, microfluidics represent a powerful tool in mimicking tissue structures requiring the presence of vascular networks.

Over the decade, various techniques have been established to pattern microfluidic networks for vasculature generation. Table 3 summarizes the development of several microfluidic vasculature models. The most common and well established fabrication method is photolithographic moulding of PDMS. Microvessels of rectangular cross-section can be easily fabricated to be later populated by human vascular endothelial cells [133]. However, the diameter of these microvessels is limited by the resolution of soft lithography. Most microvessels patterned via soft lithography are able to mimic arteries and veins of more than 100 μm in diameter [42,89,134], but are far from reaching the geometry of very fine capillaries (10–20 μm) [135]. In addition, most of the lithography-moulded microvessels are made of synthetic polymers including PDMS and are lined with a single layer of vascular endothelial cells. Such synthetic and monolayer structures are unable to mimic the natural architecture of blood vessel walls, which usually consist of stacked layers of endothelial cells, a basement membrane, pericytes, smooth muscle cells and fibroblasts. These stromal layers outside vascular endothelium are known to be very important for endothelial junction performance [136,137]. To mimic the stroma surrounding blood vessels, alternative techniques such as injection moulding have been developed to pattern vasculature inside ECM hydrogels. For instance, Baker *et al*. injection-moulded gelatin, a natural thermoplastic protein, into a microvasculature construct and embedded it within collagen hydrogel [35]. The gelatin was then melted at 37°C and left open channels inside the collagen gel. The vasculature reached a diameter of less than 100 μm and the cross-section was quasi-cylindrical. A similar strategy was developed in the same group, where 3D printed lattices made of sacrificial carbohydrate glass were used to pattern vasculature inside ECM [138]. The glass lattices were encapsulated within various ECM materials along with living cells and later dissolved in cell media to create perfusable vascular networks. These microvessels had cylindrical lumen and a minimum diameter of around 200 μm. It is worth mentioning that compared with lithography-moulded planar microchannels, such cylindrical lumen closely mimics the anatomy and hemodynamics of real blood vessels [139]. Alternatively, viscous finger patterning [140], removable nitinol rods [141] and laser beams [142] were also used to pattern cylindrical microchannels inside ECM gels. For example, Hasan *et al*. inserted concentric needles of varying diameters into a microchannel and subsequently loaded cell laden gelatin methacryloyl (GelMA) hydrogel into the annular interneedle space. The use of concentric needles was to create multilayered cylindrical vascular walls consisting of endothelial, smooth muscle and fibroblast layers [143]. A much easier approach to create ‘do-it-yourself’ endothelialized microfluidics was also reported in which researchers used removable polymethylmethacrylate optical fibers to pattern vasculature for the study of endothelial–blood cell interaction [144].

**Table 3.** **Characteristics of microfluidic vasculature generation technologies.**

**Microfluidic vasculature generation technology**	**Cross-section**	**Geometry**	**Note**	**Advantages**	**Ref.**
Photolithographic moulding	Planar	>30 μm	Most developed and standardized technology	Fluid Chemical gradient Coculture	[42]
Injection moulding	Quasi-cylindrical	>20 μm	Template molded in sacrificial material	Fluid Chemical gradient Coculture	[35]
Direct-write assembly	Cylindrical	200–800 μm	Template direct-printed from sacrificial material	Fluid Chemical gradient Coculture	[138]
Viscous finger patterning	Cylindrical	100–700 μm	Less controllable vessel geometry	Fluid Chemical gradient Coculture	[140]
Removable rods	Cylindrical	150 μm	Stiff hydrogel to withstand rod removal	Fluid Chemical gradient Coculture	[141]
Laser micromachining	Cylindrical	8 μm	Precise control over small geometry	Fluid Chemical gradient Coculture	[142]

Microfluidic vasculature mimics the geometry of vascular networks and allows for simulated body fluids, gradient and cell coculture. Various techniques have been reported to pattern microfluidic networks for vasculature generation.

Using these aforementioned microfluidic fabrication methods, microchannel networks in the hundreds of micron scale can be patterned within synthetic or natural polymeric materials and be lined with vascular endothelial cells. This microvessel generation technique can be termed as ‘prevascularization technique’ [145]. However, to replicate the complex networks of fine capillaries, angiogenesis-assisted vascularization is a more appropriate strategy. Briefly, vascular endothelial cells are seeded inside ECM hydrogels with angiogenic stimuli such as shear stress and vascular endothelial growth factor (VEGF) [130], and are cultured for days to form spontaneously interconnected capillary networks. The main limitation of the angiogenesis technique is that the system often requires a set of predefined culture conditions, which restrict system tunability and hemodynamic controllability. Table 4 cross-compares the two vascularization approaches. It is of note that individual [43] or combined [146] strategies can be employed to address various research questions. To improve physiological relevance, important directions for the future development of biomimetic vasculature include: improving coculture complexity, refining vasculature dimension and enhancing fluidic control. Another issue is system validation. Although microfluidic microvasculature systems possess great potential for studies on hemodynamics and endothelial–blood cell interaction, very few of these devices have been directly validated against *in vivo* models. More cross-system comparisons need to be completed to encourage wide applications of vasculature-on-chip.

**Table 4.** **Principle, advantages and disadvantages of two vasculature generation strategies.**

**Vasculature generation strategy**	**Principle**	**Advantages**	**Disadvantages**
Prevascularization	Endothelial lining of predefined vasculature	Precise control over vasculature architecture Precise control over flow	Simple geometry Low resolution
Angiogenesis-assisted vascularization	Chemical-induced angiogenesis in endothelial-embedded hydrogel	Complex geometry, dense network Cylindrical lumen Self-organized capillaries	Limited to tumor vasculogenesis Low controllability over flow Slow formation of vasculature

Prevascularization refers to the lining of vascular endothelial cells in the lumen of patterned microvasculature, suitable for mimicking veins and arteries of more than 50 μm in diameter. Angiogenesis-based vascularization refers to spontaneous capillary (diameter <20 μm) formation inside extracellular matrix hydrogels.

## Desirable culture features: a research survey analysis from the end user standpoint

Although a plethora of microfluidic-based culture models has been developed, as described above, the adaptation of these models to address biologically focused research questions is sparse. Among the biological community, which is the intended end user of 3D culture systems, a sceptical attitude still exists: potential customers are concerned about the reliability, functionality and reproducibility of engineered tissues. To better appreciate the expectations and concerns of biomedical researchers, we designed a comprehensive survey to assess the acceptance of 3D *in vitro* culture systems, focused on microfluidics. The questionnaire is shown as www.example.com Supplementary Data 1. It includes 12 single choice questions investigating researchers’ background and acceptability of 3D culture models, five multiple choice questions on their preferences and suggestions, and one open question on the signaling pathways they are interested in. We invited via email 70 bioscience researchers from academia and the pharmaceutical industry, to participate in the survey. The participants were not filtered by their familiarity of the authors or by certain research fields. Among the invited participants, 46 were world-renowned bioscientists who have published highly cited articles (from 2006 to date) in 15 top bioscience journals such as *Nature Biotechnology*, *Journal of Cell Biology*, *Cell* and *Nature Reviews Molecular Cell Biology*. Another 24 lab-based biomedical researchers including PhD students and early-career Postdocs (mainly based in the University of Cambridge) were also involved in the survey.

**Figure 3.**
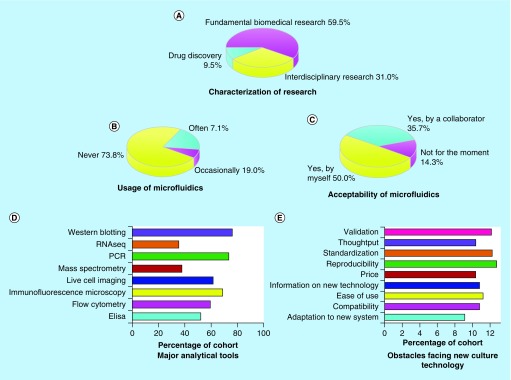
**Characterization of research area, usage and acceptability of microfluidic culture systems, usage of analytical tools and concerns about new culture technology among biomedical researchers.** **(A)** The cohort consists of scientists from fundamental, interdisciplinary research and drug discovery industry. **(B)** Few researchers have previous experience of using complex 3D culture models including microfluidics. **(C)** Acceptance of 3D culture systems including microfluidics is a predominant response. **(D)** Popular analytical tools for biomedical scientists include western blotting, PCR, immunofluorescence microscopy, live cell imaging, flow cytometry and ELISA. **(E)** Potential users are concerned about the reproducibility, standardization, validation, user-friendliness and compatibility of new culture technology. PCR: Polymerase chain reaction.

In total, 42 researchers completed the questionnaire which translates to a response rate of 60%. The participants contain a combination of 15 research group leaders, 24 lab-based researchers and three industrial scientists (see Figure 3A). Their research covers areas including cancer, neuroscience, stem cell, toxicology, endocrinology and aging. Signaling molecules and pathways such as p53, Rho GTPases, integrin, EGFRs, ROS, WNT, IR, HIF-1 are indicated as a subject of interest. Further, major analytical tools used by these researchers were investigated to identify which type of downstream assays a culture system should be able to adapt to. The data revealed that typical analytical methods employed by biomedical researchers include western blotting, polymerase chain reaction (PCR), immune-fluorescent microscopy, live cell imaging and flow cytometry (Figure 3D). When it comes to the usage of 3D culture systems such as microfluidic chips, the majority of the researchers did not have previous experience. One out of five used microfluidics occasionally and only 7.1% were familiar with this technique (Figure 3B). However, 85.7% (36 out of 42) of the researchers expressed a clear interest in adopting microfluidic culture in their research, either performed by themselves (50%) or by collaborators (35.7%) such as a provider company or a collaborative research group (Figure 3C). When queried about key obstacles that may prevent the uptake of new cell culture technologies, researchers listed five major concerns as system reproducibility, standardization, validation, ease of use and compatibility with analytical assays (Figure 3E), which should be overcome by future 3D models. However, we would like the reader to note that due to the relatively small size of the cohort, there is a possibility that this study may not capture all the opinions of researchers from the different fields.

As aforementioned, an ideal 3D culture model should demonstrate considerable microenvironment complexity to achieve a close resemblance to natural tissues. Therefore, details of desirable microenvironment features determined by biomedical researchers were summarized in Supplementary Figure 1. We classified two subgroups in the cohort: the ‘advanced-career researchers (ACR)’ group (18 out of 42), referring to scientists who have more than 7 years of research experience in their fields; and the ‘early-career researchers (ECR)’ (24 out of 42), referring to researchers with less than 7 years of experience. This classification is for the purpose of generating a detailed observation on the preference of scholars within these two groups. In total, eight microenvironment factors were evaluated and the importance of each factor was weighed using a scoring scale of 0–4 (Supplementary Figure 1A), where 0 was classified as ‘not important at all’, and 4 as ‘absolutely essential’. The data suggested that both subgroups agree on the importance of the spatial organization of cells/ECM in a 3D culture system; whereas varying views were recorded for the remaining seven features. For instance, 88.88% of the ‘ACR’ group scored above 3 (‘very important’ to ‘absolutely essential’) for the ‘coculture’ factor, while only 50% of the ‘ECR’ group expressed such a strong preference. This difference reflected a variance in researchers’ actual need and expectation for new culture models, which may influence their final decision making as well as the design priority and marketing strategy of 3D culture developers. The average weight of each microenvironment factor was listed in Supplementary Figure 1B, with spatial organization, coculture and ECM ranked as essential characteristics (scored around or above 3), while external force stimuli, topography, fluids, hypoxia and chemical gradient as desirable features (scored between 2 and 3).

**Figure 4.**
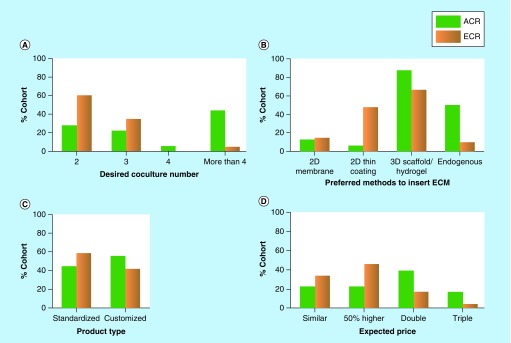
**coculture number, extracellular matrix, product type and price expected by researchers.** **(A)** ACR prefer complex 3D culture systems enabling the coculture of more than four types of cells. ECR require two or three cell types in coculture. **(B)** ACR prefer 3D scaffold/hydrogel and endogenous ECM. ECR prefer thin coating of ECM molecules and 3D scaffold/hydrogel. **(C)** ACR prefer more customized culture systems. ECR require standardized products. **(D)** ACR are willing to pay double or even triple price for 3D culture models. ECR will pay less. ACR: Advanced-career researcher; ECM: Extracellular matrix; ECR: Early-career researcher.

Further details of researchers’ preference on other 3D culture characteristics were shown in Figure 4. The two subgroups have varying views on the choice of coculture number, ECM, product type and price. For instance, half of the ‘ACR’ group desired a complex system that is able to coculture at least four types of cells, while a greater percentage (60%) of the ‘ECR’ expected a simpler system enabling only two types of cells in coculture (Figure 4A). As for the preferred method to incorporate ECM, a majority of the ‘ACR’ group opted for 3D fibrous scaffolds, hydrogel matrices and endogenous ECM, while half of the ‘ECR’ favored a simpler, 2D thin coating of ECM components (Figure 4B). Similarly, the ‘ACR’ preferred more customized, complex systems and were willing to pay up to three-times higher prices. By contrast, the other group showed greater interest in standardized models and expected lower prices (Figure 4C & D). In summary, ‘ACR’ have higher expectations for the complexity, physiological relevance of a 3D culture system and expressed clear interest in customizable products. This variance in researchers’ decision making, even sometimes subtle, should be considered in the design and marketing of 3D culture systems.

With researchers’ high expectation for 3D culture in mind, it is worth mentioning that the time and effort needed for system installation often increases with system complexity, as does the end user input and system variability. Therefore, it is important to assess researchers’ requirement on the installation, usage and performance of 3D culture systems. Our data revealed that 61.9% (26 out of 42) of the researchers would like to see the completion of the preliminary training and setting up of a 3D culture platform within 4 weeks (Supplementary Figure 2A). Approximately half of the cohort expected similar or higher success rate in the new systems, in comparison to the traditional 2D systems. However, this expectation is currently difficult to achieve because 3D culture has not yet entered its maturity stage. For instance, many of the complex 3D culture models often fail to reach expected levels of robustness and reproducibility. With this in mind, 28.6% of the cohort accepted a 20% decrease in success rate and 21.4% accepted a 50% decrease (Supplementary Figure 2B). As to the preparation time, more than one-third of the researchers expected to have a 3D culture device made within 3 days, while a greater percentage (45.2%, 19 out of 42) are willing to wait for 4–7 days (Supplementary Figure 2C). Also, it can be concluded that depending on the research purpose, the required time that cells be maintained in a 3D culture device varies from days to weeks (Supplementary Figure 2D).

Overall, willingness to adopt 3D culture was the prominent response among biomedical researchers. Key microenvironment features desired by researchers are spatial organization, ECM and coculture of heterotypic cells. Other desirable factors, depending on the research question to address, include external force stimuli, topography, simulated body fluids, hypoxia and chemical gradients. These results resonate with experts’ opinions expressed in other reviews on 3D culture [147–150]. In addition, we observed slight variance in the decision making of end users in that ‘ACR’ opted for 3D models with higher coculture ability, closer physiological relevance and were willing to pay higher prices for customized models. Despite this positive attitude, our survey implied that there still exists a significant gap between the model complexity demanded by especially the ACR, and the capability demonstrated in existing culture systems (as summarized in the previous sections). Although current microfluidic chips, including some commercialized ones such as OrganoPlates, offer a convenient system that to some extent mimics *in vivo* conditions, few studies using these culture models have been published in leading journals in fundamental biomedical research or pharmaceutical science. Most engineered culture systems are able to demonstrate spatial organization in their biomimetic architectures but lack comprehensive downstream physiological or pharmacological analysis that can fully validate their functionality. Some exceptions do exist, as illustrated in the recent work by Soroush *et al*., in which a microfluidic vascular network based on *in vivo* images revealed a key role of protein kinase C δ in neutrophil transendothelial migration [79]. However, a more ‘killer application’ may need to be showcased to stimulate a broader uptake of 3D culture models. We believe that achieving a critical set of ‘baseline’ complexity desired by the biomedical researchers, simultaneously with improved system reproducibility, functionality and user friendliness are the critical stepping stones toward the broader adoption of 3D culture technologies. Otherwise, there is not a strong enough incentive for the biological community to modify their culture protocols and invest in a new, nonstandardized platform. This hesitation may be a hurdle for new culture systems to be accepted as valuable tools for fundamental biomedical research and drug screening in an industrial context.

## Conclusion

The versatile functionality and excellent spatiotemporal control over microenvironmental elements in 3D cell culture opens up wide possibilities for tissue engineering and next generation drug testing. Specifically, the ability of microfluidics to biomimic the fluidic *in vivo* microenvironment and micron-scale luminal structures can be a powerful tool in the creation of organ/vasculature-on-chips. According to our research questionnaire, willingness to adopt 3D culture technology was a prominent response among biomedical researchers, although a significant gap still exists between the desired systems and existing 3D culture options. The survey may provide academic insight for entrepreneurs who are interested in the commercialization of microfluidic-based 3D culture systems.

## Future perspective

The past two decades have seen the emergence and rapid growth of microfluidics-based 3D culture technology, with the ultimate aim to boost the development of fundamental bioscience and pharmaceutics. A plethora of interesting microfluidic models have been engineered, such as the biomimetic organ- and vasculature-on-a-chip. Despite the many advantages microfluidics offer, they are not without weaknesses. The miniaturization empowered by microfluidics also implies that accessible cells/tissues in the culture system can be too few to be further studied using conventional analytical tools, such as western blotting, ELISA and mass spectrometry. Although some on-chip assays including single-cell PCR [151] and western blotting [152] have been developed for the small sample input from microfluidics, samples in current biomimetic chips are mostly studied using 2D/3D imaging techniques including epifluorescence and confocal microscopy. However, the wide adaptation of microfluidics-based culture methods requires that the systems be validated from a multidimensional angle, by various analytical tools. Therefore, either the culture models should be designed more compatible with existing analytical methods, or more sensitive, advanced analytical/imaging tools have to be developed for or even integrated in microfluidic chips [153]. In addition, most microfluidic systems, compared with the conventional petri dishes, are far less user friendly. Although standardized protocols are now available [42,154], microfluidic systems are still labor-intensive to fabricate and complex to operate. For example, microfluidics often require specific training on the pipetting/coating techniques as well as complicated external setups such as syringe pumps. It is therefore desirable but still challenging that future microfluidics incorporate intelligent on-chip control of oxygen/medium perfusion, while user operation is simplified at the same time.

Though creating a fully functional artificial organ may seem too ambitious, tissue engineers could work toward achieving a decent level of complexity, both structurally and functionally, and from micro to nano scale, in one biomimetic culture system. To realize more physiologically relevant tissue architecture and microenvironment dynamics, microfluidics should explore new fabrication materials beyond PDMS [155], and be combined with other bioengineering tools such as nano-fibrous scaffolds, biosensors and 3D printing [156]. However, for tissue engineering scientists, developing cutting-edge technology for 3D culture is no longer the only focus. The bioengineering community should reach out proactively to their biomedical end users, to seek their mutual interests, finding design inspiration from their feedback.

Encouragingly, our research survey revealed that a positive attitude toward 3D culture prevails among biomedical scientists and pharmaceutical researchers. In fact, the past decade saw many encouraging attempts to apply 3D culture in areas such as cancer metastasis research [157–159] and early-phase drug screening [160]. For instance, a microfluidic device with interconnected chambers housing liver, tumor and marrow cells was used to test the metabolism-dependent cytotoxicity of an anticancer drug, Tegafur [161]. Other applications of microfluidic culture can be found in neuroscience, for purposes of complementing *in vivo* studies on neuronal signaling [162] and high throughput evaluation of neurotoxic compounds [100]. These examples showed how microfluidics-based culture systems can help to bridge the gap between *in vitro* and *in vivo* study. As such, we believe that a lot more exciting research with impactful clinical effect can be performed when 3D culture systems are employed in broader areas. For instance, microfluidics-based 3D culture can be further applied for the culture of patient-derived biopsy, for the purpose of developing personalized therapy. Microfluidics also represents a desirable tool to help test our understanding on human stem cell niches, organ development and regenerative medicine. However, in order to become a widely accepted tool in fundamental bioscience and pharmaceutical industry, 3D culture models have to find suitable research questions to address and impart tailored complexity, while overcoming drawbacks such as poor compatibility, relatively low throughput, limited functionality and lack of a standardized metric in cross-system comparison.

**Executive summary**

• An ideal 3D culture system should demonstrate a balance of complexity, user-friendliness, physiological relevance and controllability.
• Microfluidics is a valuable tool for the development of organ- and vasculature-on-a-chip.
• Willingness to adopt 3D culture technology was a prominent response among biomedical researchers, although a significant gap still exists between the desired systems and existing 3D culture options.
