# Supplementary material for: Microfluidic on-chip biomimicry for 3D cell culture: a fit-for-purpose investigation from the end user standpoint
Source: Future Sci OA. 2017 Mar 2;3(2):FSO173. doi: 10.4155/fsoa-2016-0084 (PMC5481809; doi:10.4155/fsoa-2016-0084)
Supplement: Supplementary file 1 [file fsoa-03-173-s1.docx]

Research survey on microfluidics-based 3D culture systems

1. You are currently working in

- Academia
- Industry

2. Please select the characteristic of your research

- Interdisciplinary
- Drug discovery/ therapeutic
- Fundamental medical/ biological research

3. Please indicate your field of research (Multiple choice possible)

- Aging
- Cancer
- Endocrinology
- Neuroscience
- Stem cell
- Toxicology

4. What types of analysis do you perform in your study? (Multiple choice possible)

- Elisa
- Flow cytometry
- Immunofluorescence microscopy
- Live cell imaging
- Mass spectrometry
- PCR
- RNAseq
- Western blotting

5. Is the spatial organisation of cells important for your study?

- Definitely yes
- Probably yes
- Probably not
- Don't know

6. If technology allowed, what are the desired features for your cell culture system? (Please give points to the following factors)

|  | 0 = Not important at all | 1 = Of little importance | 2 = Of average importance | 3 = Very important | 4 = Absolutely essential |
| --- | --- | --- | --- | --- | --- |
| Chemical gradient |  |  |  |  |  |
| Co-culture of different cell types |  |  |  |  |  |
| External force stimuli |  |  |  |  |  |
| ECM |  |  |  |  |  |
| Hypoxia |  |  |  |  |  |
| Topography |  |  |  |  |  |
| Simulated blood flow or interstitial fluids |  |  |  |  |  |

6.1. How many type of cells would you like to co-culture?

- 2
- 3
- 4
- > 4

6.2. How would you like to incorporate ECM into your culture system? (Multiple choice possible)

- 2D thin coating of ECM molecules or hydrogel
- 2D ECM membrane
- 3D ECM scaffold or hydrogel
- Endogenous ECM

7. Have you used microfluidics for 3D cell culture?

- Never
- Occasionally
- Often

8. Would you consider using a complex 3D cell culture system like microfluidics?

- Yes, by myself or by a member of my group/ company if some supports and literature are available
- Yes, but only if the technology is fully supported by a collaborator or a service company
- Not for the moment

9. How much time would you be willing to spend in the preliminary training and setting up of a complex 3D microfluidic culture system?

- Less than 1 week
- 1-4 weeks
- 1-2 month
- Up to 1 year as long as it gives me the desired results

10. Once set up, how much time do you expect to spend on preparing one microfluidic cell culture sample?

- Less than 3 days
- 4-7 days
- 1-2 weeks

11. For how long would you like to maintain one microfluidic cell culture sample in you study?

- < 3 days
- 3 to 7 days
- 1 to 3 weeks
- More than 3 weeks

12. What success rate would you be willing to compromise compared to your current culture model, for your desired complex culture system?

- Similar or better success rate
- Up to 20% decrease in success rate
- Up to 50% decrease in success rate
- Up to 70% decrease in success rate

13. Do you prefer a standardized or a customized culture system?

- Standardized
- Customized

14. In comparison to your current culture methods, at what price would you be willing to pay for a complex 3D cell culture system?

- Similar price
- Up to 50% higher in price
- Up to 100% higher in price
- Up to 3 times higher in price
- Up to 10 times higher in price

15. What signaling pathway are you interested in?

- Confidential/not applicable
- The pathway I am interested in is ____________________

16. What do you think are key obstacles preventing the uptake of new cell culture technologies? (Please give points to the following factors)

|  | 0 = Not important at all | 1 = Of little importance | 2 = Of average importance | 3 = Very important | 4 = Absolutely essential |
| --- | --- | --- | --- | --- | --- |
| Adaptation to a new system |  |  |  |  |  |
| Compatibility with your existing biochemical analyses |  |  |  |  |  |
| Ease of use |  |  |  |  |  |
| Information available about the new technology |  |  |  |  |  |
| Price |  |  |  |  |  |
| Reproducibility |  |  |  |  |  |
| Standardisation |  |  |  |  |  |
| Though-put |  |  |  |  |  |
| Validation |  |  |  |  |  |
