# Supplementary material for: Microfluidic on-chip biomimicry for 3D cell culture: a fit-for-purpose investigation from the end user standpoint
Source: Future Sci OA. 2017 Mar 2;3(2):FSO173. doi: 10.4155/fsoa-2016-0084 (PMC5481809; doi:10.4155/fsoa-2016-0084)
Supplement: Supplementary file 2 [file fsoa-03-173-s2.docx]

#
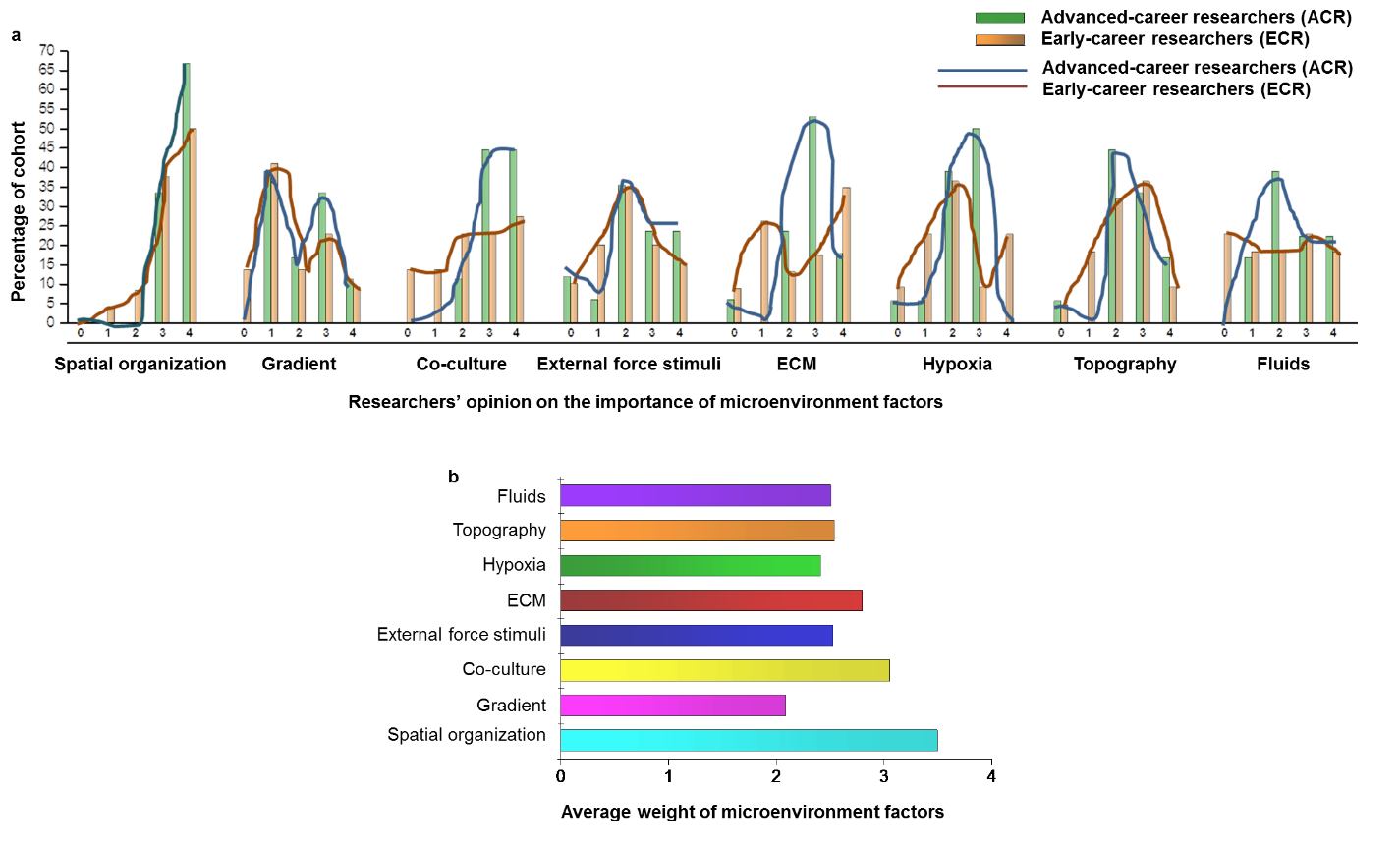


**Supplementary Figure 1.** Desirable microenvironment features determined by biomedical researchers. (a) The importance of 8 microenvironment factors was weighed by researchers using a scoring scale of 0-4 (0 “not important at all”, 1 “of little importance”, 2 “of average importance”, 3 “very important” and 4 “absolutely essential”). Variances in the weight distribution were observed between the two subgroups. (b) Average weight of each microenvironment features showed that spatial organization, co-culture of heterotypic cells, ECM are essential microenvironment characteristics (scored around or above 3) for 3D cell culture.
