# Supplementary material for: Microfluidic on-chip biomimicry for 3D cell culture: a fit-for-purpose investigation from the end user standpoint
Source: Future Sci OA. 2017 Mar 2;3(2):FSO173. doi: 10.4155/fsoa-2016-0084 (PMC5481809; doi:10.4155/fsoa-2016-0084)
Supplement: Supplementary file 3 [file fsoa-03-173-s3.docx]

**
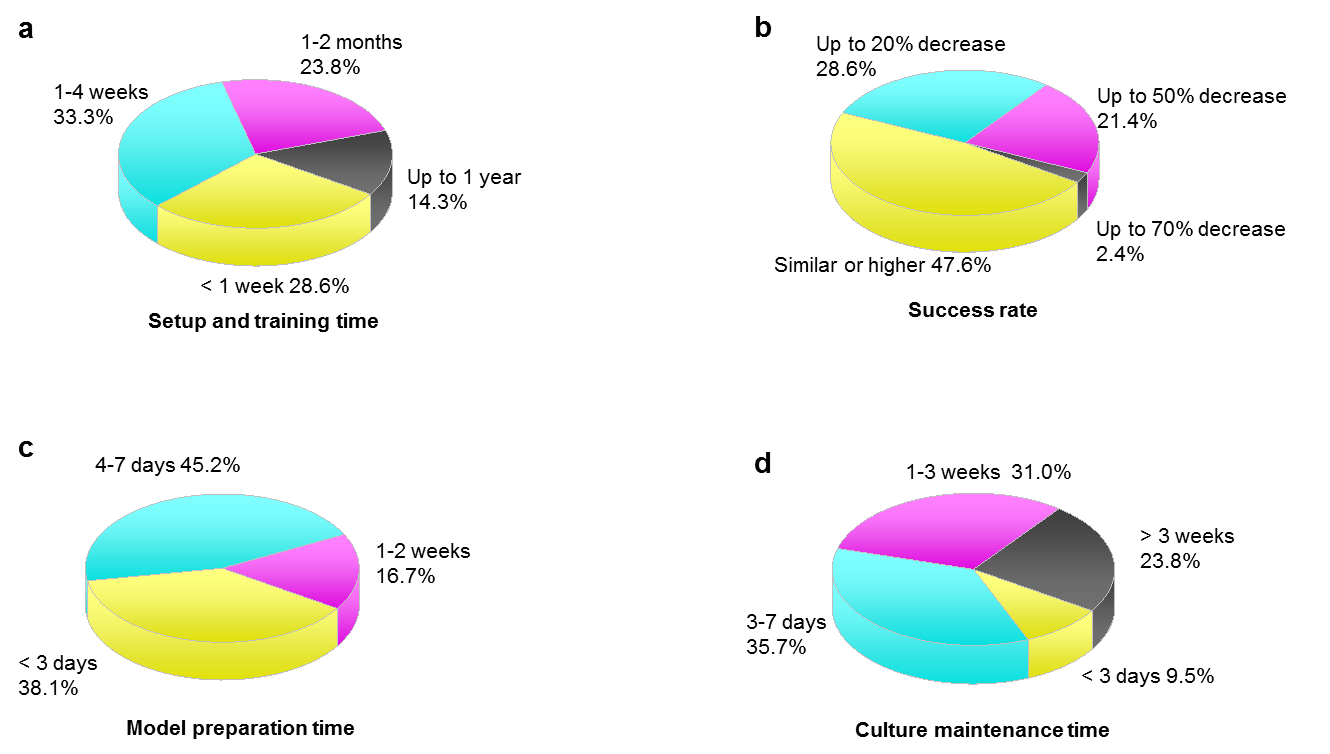
**

**Supplementary Figure 2.** Expected installation, performance and usage of 3D culture systems. (a) The majority of researches expected the initial training and setup of a 3D culture system complete within 1 month. (b) Researchers tolerate up to 50% decrease in the success rate of a 3D culture system. (c) Expected preparation time for each model is less than 1 week. (d) Systems are to be maintained for more than 3 days.
